# Effect of Bronchodilator and Steroid Use on Heart Disease and Stroke Risks in a Bronchiectasis–Chronic Obstructive Pulmonary Disease Overlap Cohort: A Propensity Score Matching Study

**DOI:** 10.3389/fphar.2019.01409

**Published:** 2019-11-27

**Authors:** Jun-Jun Yeh, Yu-Cih Yang, Chung Y. Hsu, Chia-Hung Kao

**Affiliations:** ^1^Department of Family Medicine, Ditmanson Medical Foundation Chia-Yi Christian Hospital, Chiayi, Taiwan; ^2^Department of Early Childhood Education and Nursery, Chia Nan University of Pharmacy and Science, Tainan, Taiwan; ^3^College of Medicine, China Medical University, Taichung, Taiwan; ^4^Management Office for Health Data, China Medical University Hospital, Taichung, Taiwan; ^5^Graduate Institute of Biomedical Sciences and School of Medicine, College of Medicine, China Medical University, Taichung, Taiwan; ^6^Department of Nuclear Medicine, China Medical University Hospital, Taichung, Taiwan; ^7^Department of Bioinformatics and Medical Engineering, Asia University, Taichung, Taiwan; ^8^Center of Augmented Intelligence in Healthcare, China Medical University Hospital, Taichung, Taiwan

**Keywords:** heart disease, stroke, bronchiectasis–chronic obstructive pulmonary disease overlap syndrome, bronchodilator, steroid

## Abstract

**Background:** To determine the effects of bronchodilator, steroid, and anti-arrhythmia drug use on the risk of heart disease/stroke (HDS) in patients with bronchiectasis–chronic obstructive pulmonary disease overlap syndrome (BCOS).

**Methods:** We retrospectively enrolled patients with BCOS (BCOS cohort, n = 1,493) and patients without bronchiectasis and chronic obstructive pulmonary disease (COPD) (non-BCOS cohort, n = 5,972). The cumulative incidence of HDS was analyzed through Cox proportional regression. We calculated adjusted hazard ratios (aHRs) and their 95% confidence intervals (CIs) for HDS after adjustments for sex, age, comorbidities, long-acting β2-agonist or long-acting muscarinic antagonist (LABAs/LAMAs) use, short-acting β2-agonist or short-acting muscarinic antagonist (SABAs/SAMAs) use, oral steroid (OSs) or inhaled corticosteroid steroid (ICSs) use, and anti-arrhythmia drugs use.

**Results:** The aHR (95% CI) for HDS was 1.08 (0.28–4.06) for patients using LAMAs compared with those not using drugs. Regarding drug use days, the aHRs (95% CIs) were 32.2 (1.79–773.0), 1.85 (1.01–3.39), and 31.1 (3.25–297.80) for those with recent SABAs use, past ICSs use, and past anti-arrythmia drugs use, respectively. Regarding cumulative drug dose, the aHRs (95% CIs) were 2.12 (1.46–3.10), 3.48 (1.13–10.6), 3.19 (2.04–4.99), 28.1 (1.42–555.7), 2.09 (1.32–3.29), 2.28 (1.53–3.40), and 1.93 (1.36–2.74) for those with a low dose of SABAs, medium dose of SABAs, low dose of SAMAs, low dose of ICSs, medium dose of ICSs, low dose of OSs, and medium dose of OSs, respectively.

**Conclusions:** Compared with patients without bronchiectasis and COPD, BCOS patients with recent SABAs, past ICSs, and past anti-arrhythmia drugs use; a low or medium SABAs ICSs, and OSs dose; and a low SAMAs dose had a higher risk of HDS. LAMAs were not associated with HDS.

## Introduction

The prevalence of cardiovascular disease and stroke is increasing across the globe, as is the burden of these diseases (Garcia et al., 2016; Benjamin et al., 2018). Chronic obstructive airway diseases include chronic obstructive pulmonary disease (COPD) and bronchiectasis. According to the 2015 Global Burden of Disease Study (GBD 2015 Chronic Respiratory Disease Collaborators, 2017), COPD caused 3.2 million deaths worldwide in 2015. COPD is characterized by airflow limitation that is usually progressive and associated with persistent small airway inflammation, and it is a major risk factor for heart disease/stroke (HDS). A relatively recent study revealed that COPD with acute exacerbations is associated with an increased risk of cardiac arrhythmia with sudden death (Leitao Filho and Sin, 2018).

Bronchiectasis is a chronic respiratory disease characterized by a clinical syndrome of cough, sputum production, and bronchial infection, in addition to abnormal and permanent bronchial dilatation (Polverino et al., 2017; Hung et al.,2018). Because of the increased use of high-resolution computed tomography (HRCT;Prendki et al.,2018; Yeh, 2018c; Yeh, 2019a), the diagnostic sensitivity for bronchiectasis has increased (Schäfer et al., 2018). The clinical presentation of bronchiectasis overlaps with that of other respiratory disorders, such as asthma and COPD (Martinez-Garcia and Miravitlles, 2017). For example, Polverino et al. (2018) examined computed tomography (CT) images obtained for patients and reported that 4%–72% of patients with severe COPD had radiological bronchiectasis; they also reported that 20%–30% of patients with severe or uncontrolled asthma had bronchiectasis.

A single-center study demonstrated an approximately three fold increase in the mortality rate of patients with bronchiectasis; specifically, patients with bronchiectasis and COPD had a 5-year mortality rate of 55% compared with the rate of 20% in those with bronchiectasis without COPD (Hurst et al., 2015). Patients with bronchiectasis and COPD were also found to have an increased rate of acute respiratory events (Chung and Lin, 2018). Thus, bronchiectasis–COPD overlap syndrome (BCOS) may be considered a separate disorder (Chalmers, 2017; Poh et al., 2017). Treatments successful in managing COPD may not be as effective at managing bronchiectasis (and vice versa); this phenomenon is best exemplified by inhaled corticosteroids (ICSs). ICSs are widely used in COPD treatment but are not recommended for most patients with bronchiectasis. By contrast, inhaled antibiotics, including anti-pseudomonal agents in appropriate patients, are beneficial and appear in current bronchiectasis guidelines but are not used routinely in treating stable COPD (Hurst et al., 2015).

Studies have yet to evaluate the effect of bronchodilator and ICSs/oral steroid (OSs) use on the risk of HDS in patients with BCOS. Current knowledge holds that the frequency of arrhythmias such as atrial fibrillation is high—up to 29%—in patients with COPD (Grymonprez et al., 2019) and that anti-arrhythmia drugs play a role in the treatment of COPD with HDS (Trinkmann et al., 2019). Therefore, in the present study, we investigated the effect of bronchodilator, ICSs/OSs, and antiarrhythmic drugs use on the risk of HDS in a cohort of patients with BCOS in comparison with a cohort of patients with COPD alone.

## Methods

### Data Source

We used the Longitudinal Health Insurance Database 2000 (LHID2000), a subset of the National Health Insurance Research Database (NHIRD) established by the National Health Research Institutes (NHRI) in Taiwan. The NHRI reported no significant differences in the distribution of sex or age among beneficiaries in the LHID2000; thus, the database can be considered representative of the general population. The LHID2000 contains comprehensive information, including demographic data, all records of outpatient and inpatient hospital visits, complete prescription details, and diagnostic codes based on the International Classification of Diseases, Ninth Revision, Clinical Modification (ICD-9-CM). For privacy protection, the personal identification numbers of all insurants were scrambled cryptographically to ensure anonymity before the data were released for research purposes. This study was exempted from a review by the Internal Review Board of China Medical University and Hospital (CMUH104-REC2-115-CR4).

### Sample Patients

To determine the prevalence of HDS in patients with bronchiectasis (ICD-9-CM code 494) and COPD (ICD-9-CM codes 491, 492, and 496), we selected a cohort of patients with BCOS who had two outpatient visits or an inpatient visit for BCOS and selected a comparable control cohort of patients without BCOS. For inclusion in the BCOS cohort, BCOS had to have been diagnosed between January 1, 2000, and December 31, 2012. ses of atients without BCOS was randomly selected from LHID2000 enrollees without any history of BCOS at a selection ratio of 1:1 based on 5-year intervals and according to sex, index year, sleep disturbance, pulmonary tuberculosis, pneumonia, asthma, diabetes mellitus, hypertension, hyperlipidemia, pulmonary embolism, and medication use (including use of LABAs, LAMAs, SABAs, SAMAs, ICSs, OSs, and anti-arrhythmia drugs) through propensity score matching. Those in the comparison cohort were paired with matching patients, and a month and day in the same year were randomly assigned as their index dates. Patients with a history of HDS before the index date were excluded from both study cohorts. All patients were followed up until they developed HDS, they died, they withdrew from the NHI program, or December 31, 2013, whichever occurred first.

### Outcome and Medication

The primary outcome of this study was an individual event of heart disease (ICD-9-CM codes 410–414 and 425–429) or stroke (ICD-9-CM codes 433–434, 435, and 436). Data for underlying comorbidities, namely sleep disturbance (ICD-9-CM code 7805), pulmonary tuberculosis (PTB, ICD-9-CM codes 010–012), pneumonia (ICD-9-CM codes 481–483 and 485–486), asthma (ICD-9-CM code 493), diabetes mellitus (DM, ICD-9-CM code 250), hypertension (ICD-9-CM codes 401–405), and pulmonary embolism (ICD-9-CM code 415.1), were also collected. Furthermore, this study evaluated the effect of several drugs used for BCOS treatment on the risk of HDS, namely LABAs (Anatomical Therapeutic Chemical [ATC] codes R03AC12 and R03AC13), LAMAs (ATC code R03BB04), SABAs (ATC codes R03AC02, R03AC03, and R03AC04), SAMAs (ATC code R03BB01), ICSs (ATC codes R03BA01, R03BA02, R03BA05, and R03BA08), OSs (ATC codes D07AC15, R01AD05, H02AB02, D07AC17, S01BA02, H02AB04, H02AB06, H02AB08, and S01BA02), and anti-arrhythmia drugs (ATC code C01B). We accounted for several confounding factors, such as age, sex, comorbidities, and medication use.

### Statistical Analysis

For each patient with BCOS, a matched control was assigned using propensity score matching to account for baseline differences between patients with and without BCOS. Demographic characteristics and the prevalence of comorbidities were compared using standardized mean difference, for which values of ≤0.10 were considered to indicate a negligible difference between two cohorts. We estimated the cumulative incidence of HDS for both the BCOS and comparison cohorts by using the Kaplan – Meier method, and we examined the difference between the two curves by using the log-rank test. The Cox regression model was used to calculate the adjusted hazard ratios (aHRs) and 95% confidence intervals (CIs) for comparing the relative risk of HDS in the BCOS and non-BCOS cohorts. Comorbidities and drug use were included as covariates to account for potential confounding effects. We also analyzed the drug use days before HDS incidence to determine the effect of duration and dose on the risk of HDS in the BCOS cohort. Drug use days were categorized into the following subgroups: current use (drug use within 30 days of HDS incidence), recent use (drug use 30–90 days before HDS incidence), and past use (drug use >90 days before HDS incidence). We used SAS software (version 9.4 for Windows; SAS Institute, Cary, NC) for all statistical analyses and Kaplan–Meier curves for survival analysis. A two-sided *P* value of <0.05 was considered statistically significant.

### Sensitivity Analysis

As noted in the previous section, three subgroups of drug use days (current, recent, and past) were defined. The cumulative DDD was analyzed to determine the effect of duration and dose on the risk of HDS in the BCOS cohort.

### Validation of Bronchiectasis and COPD

After observing HRCT scans, Polverino et al. (2018) determined that up to 3,521 of 7,156 patients were diagnosed as having bronchiectasis. On the basis of data from the NHIRD, Cheng et al. (2015) found that the following clinical presentations and tests were associated with a high diagnostic sensitivity for COPD: ever smoking (82.9%), cough with sputum (79%), chest X-ray (84.7%), pulmonary function testing (58.4%), and CT (39.4%). Recently, Ho et al. (2018) validated the COPD cohort identified from the NHIRD in Taiwan; they determined that factors, including spirometry testing and diagnostic ICD-9-CM codes for COPD, increased the positive predictive value to 84.6%. This suggests that the BCOS results derived from the COPD cohort in the NHIRD are representative of patients with both bronchiectasis and COPD in the general population.

## Results

After propensity score matching, we included 2,816 patients with COPD (1,412 women and 1,404 men) in our study cohort. The experimental cohort (n = 1,408) consisted of patients with BCOS (n = 1,186) and patients with both BCOS and asthma (n = 222). The control cohort (n = 1,408) consisted of patients with only COPD (n = 1,042) and patients with ACO (n = 366). The mean ages of the BCOS and non-BCOS cohorts were 59.0 ± 15.3 and 53.9 ± 20.4 years, respectively, and most of the patients were in the age range of 40–64 years. Compared with the BCOS cohort, the non-BCOS cohort had a higher proportion of patients with sleep disturbance, asthma, diabetes mellitus, hypertension, hyperlipidemia, LABAs use, SABAs use, SAMAs use, ICSs use, OSs use, and anti-arrhythmia drugs use (**Table 1**).

**Table 1:** Baseline characteristics of the study population after propensity matching.

Variables	PS-matching population				Standardized mean differences^§^
	Bronchiectasis-with COPD group (n = 1,408)		Comparison group (n = 1,408)		
	N	%	N	%	
**Sex**					
female	655	46.5	757	53.7	0.14
male	753	53.5	651	46.3	0.14
**Age at baseline, year**					
<20	15	1.07	95	6.75	0.29
20–39	138	9.80	230	16.3	0.19
40–64	726	51.5	637	45.2	0.12
≥65	529	37.6	446	31.6	0.12
mean(SD)	59.01 (15.3)		53.96 (20.4)		0.28
**Comorbidity**					
Sleep disturbance	16	1.14	69	4.90	0.22
Pulmonary tuberculosis	128	9.09	162	11.5	0.08
Pneumonia	330	23.4	387	27.5	0.09
Asthma	222	15.7	366	25.9	0.25
DM	179	12.7	346	24.5	0.30
Hypertension	230	16.3	299	21.2	0.12
Hyperlipidemia	209	14.8	292	20.7	0.15
Pulmonary embolism	3	0.21	4	0.28	0.01
**Medication**					
LABAs	154	10.9	168	11.9	0.03
LAMAs	37	2.63	25	1.78	0.05
SABAs	356	25.2	510	36.2	0.23
SAMAs	261	18.5	294	20.8	0.05
ICSs	234	16.6	317	22.5	0.14
OSs	673	47.8	776	55.1	0.14
Anti-arrhythmics	27	1.92	55	3.91	0.11

COPD, chronic obstructive pulmonary disease; DM, diabetes mellitus.

^§^A standardized mean difference of ≤0.10 indicates a negligible difference between the two cohorts.

In this study, 780 patients developed HDS (**Table 2**). The incidence of HDS was higher in the patients with BCOS than in non-BCOS (5.65 and 3.26 per 100 person-years, respectively; aHR, 1.68; 95% CI, 1.45–1.94). The risk of HDS was higher in the BCOS cohort than in the non-BCOS cohort across both sex and age stratifications. Compared with patients without BCOS, patients with BCOS also had higher risk of HDS coupled with pulmonary tuberculosis (1.76-fold, 95% CI = 1.18–2.63), asthma (1.57-fold, 95% CI = 1.16–2.13), diabetes mellitus (1.57-fold, 95% CI = 1.16–2.13), hypertension (1.68-fold, 95% CI = 1.27–2.23), LABAs use (2.28-fold, 95% CI = 1.18–4.40), SABAs use (2.10-fold, 95% CI = 1.54–2.86), SAMAs use (2.73-fold, 95% CI = 1.85–4.02), ICSs use (2.57-fold, 95% CI = 1.67–3.96), and OSs use (1.85-fold, 95% CI, 1.49–2.31). By contrast, sleep disturbance, pneumonia, LAMAs use (aHR, 1.08; 95% CI, 0.28–4.06) and anti-arrhythmia drugs use (aHR, 2.78; 95% CI, 0.97–7.96) were not associated with a significantly increased risk of HDS.

**Table 2:** Incidence rate and hazard ratio of stroke or heart-disease between the two groups stratified by gender, age, comorbidities and drug use after propensity matching.

	Bronchiectasis - COPD						Crude HR (95% CI)	Adjusted HR (95% CI)
	No			Yes				
	Event	PY	IR	Event	PY	IR		
**Overall**	340	10,402	3.26	440	7,774	5.65	1.67 (1.45–1.92)***	1.68 (1.45–1.94)***
**Gender**								
Female	181	5,692	3.17	188	3,835	4.90	1.48 (1.21–1.82)***	1.57 (1.27–1.94)***
Male	159	4,710	3.37	252	3,939	6.39	1.83 (1.50–2.24)***	1.82 (1.48–2.24)***
**Age**								
< 20	0	915	0	0	148	0	–	–
20–39	25	2,160	1.15	15	1,037	1.44	1.27 (0.66–2.42)	3.47 (1.50–8.05)**
40–64	151	4,914	3.07	200	4,480	4.46	1.42 (1.15–1.76)**	1.69 (1.35–2.12)***
≥65	164	2,413	6.79	225	2,109	10.6	1.53 (1.25–1.87)***	1.54 (1.25–1.90)***
**Comorbidity**								
Sleep disturbance								
No	317	9,798	3.23	431	7,680	5.61	1.66 (1.44–1.93)***	1.71 (1.47–1.98)***
Yes	23	604	3.80	9	94	9.57	2.65 (1.22–5.76)*	0.56 (0.18–1.77)
Pulmonary tuberculosis								
No	287	9,250	3.10	380	6,978	5.44	1.68 (1.44–1.96)***	1.70 (1.45–1.99)***
Yes	53	1,152	4.60	60	796	7.53	1.61 (1.11–2.34)*	1.76 (1.18–2.63)**
Pneumonia								
No	213	7,750	2.74	308	5,825	5.28	1.83 (1.54–2.18)***	1.94 (1.62–2.34)***
Yes	127	2,652	4.78	132	1,949	6.77	1.39 (1.09–1.77)**	1.18 (0.92–1.52)
Asthma								
No	254	7,360	3.45	342	6,457	5.29	1.49 (1.26–1.75)***	1.61 (1.36–1.92)***
Yes	86	3,042	2.82	98	1,317	7.44	2.50 (1.86–3.34)***	1.57 (1.16–2.13)**
DM								
No	218	7,805	2.79	351	6,735	5.21	1.79 (1.51–2.13)***	1.68 (1.41–2.00)***
Yes	122	2,597	4.69	89	1,039	8.56	1.79 (1.36–2.35)***	1.66 (1.25–2.21)***
Hypertension								
No	234	8,102	2.88	321	6,433	4.98	1.65 (1.40–1.96)***	–
Yes	106	2,300	4.60	119	1,341	8.87	1.88 (1.44–2.44)***	1.68 (1.27–2.23)***
Hyperlipidemia								
No	227	8,128	2.79	340	6,394	5.31	1.82 (1.54–2.16)***	–
Yes	113	2,274	4.96	100	1,380	7.24	1.42 (1.09–1.86)**	1.45 (1.10–1.92)**
Pulmonary embolism								
No	339	10,376	3.26	438	7,769	5.63	1.66 (1.44–1.92)***	1.66 (1.44–1.92)***
Yes	1	26	3.84	2	5	40	–	–
**Medication**								
LABAs								
Non-use	323	8,993	3.59	404	6,854	5.89	1.58 (1.36–1.83)***	1.62 (1.39–1.88)***
Use	17	1,409	1.20	36	920	3.91	3.14 (1.76–5.62)***	2.28 (1.18–4.40)*
LAMAs								
Non-use	335	10,201	3.28	432	7,587	5.69	1.67 (1.44–1.93)***	1.68 (1.45–1.94)***
Use	5	201	2.48	8	187	4.27	2.06 (0.65–6.51)	1.08 (0.28–4.06)
SABAs								
Non-use	263	6,329	4.15	334	5,630	5.93	1.37 (1.16–1.61)***	1.56 (1.31–1.84)***
Use	77	4,073	1.89	106	2,144	4.94	2.59 (1.93–3.48)***	2.10 (1.54–2.86)***
SAMAs								
Non-use	296	8,096	3.65	353	6,281	5.62	1.48 (1.26–1.72)***	1.53 (1.31–1.80)***
Use	44	2,306	1.90	87	1,493	5.82	3.01 (2.09–4.33)***	2.73 (1.85–4.02)***
ICSs								
Non-use	302	7,714	3.91	372	6,340	5.86	1.44 (1.24–1.68)***	1.53 (1.31–1.79)***
Use	38	2,688	1.41	68	1,434	4.74	3.29 (2.21–4.90)***	2.57 (1.67–3.96)***
OSs								
Non-use	184	4,343	4.23	241	3,716	6.48	1.44 (1.19–1.74)***	1.57 (1.29–1.92)***
Use	156	6,059	2.57	199	4,058	4.90	1.89 (1.53–2.33)***	1.85 (1.49–2.31)***
Anti-arrhythmia								
Non-use	325	9,979	3.25	431	7,611	5.66	1.67 (1.45–1.93)***	1.69 (1.45–1.96)***
Use	15	423	3.54	9	163	5.52	1.58 (0.69–3.63)	2.78 (0.97–7.96)

PY, person-years; IR, incidence rate, per 100 person-years; HR, hazard ratio; CI, confidence interval.

HR was adjusted for gender, age, sleep disturbance, pulmonary tuberculosis, pneumonia, asthma, diabetes mellitus, hypertension, hyperlipidemia, pulmonary embolism, LABAs-use, LAMAs-use, SABAs-use, SAMAs-use, ICSs-use, OSs-use, and anti-arrhythmia drugs use.

––Unable to calculate because of few or no events.

*p < 0.05, **p < 0.01, ***p < 0.001.

**Table 3** presents the incidence and hazard ratios (HRs) of HDS stratified by drug use days in the BCOS and non-BCOS cohorts. Recent SABAs use, past ICSs use, and past anti-arrhythmia drugs use were significantly associated with a 32.2-fold (95% CI = 1.79–773.0), 1.85-fold (95% CI = 1.01–3.39), and 31.1-fold (95% CI = 3.25–297.8) higher risk of HDS in the BCOS cohort, respectively, compared with the non-BCOS cohort.

**Table 3:** Incidence rate and hazard ratio of stroke or heart-disease between the two groups stratified by day of drug-use after propensity matching.

	Bronchiectasis - COPD						Crude HR (95%CI)	Adjusted HR (95%CI)
	No			Yes				
	Event	PY	IR	Event	PY	IR		
**Drug-use days**								
LABAs								
Non use	327	9,031	3.62	424	6,923	6.12	1.63 (1.41–1.89)***	1.62 (1.40–1.89)***
Current use (≤30 d)	0	163	0	1	130	0.76	–	–
Recent use (30–90 d)	1	82	1.21	1	47	2.12	2.56 (0.12–52.8)	–
Past use (>90 d)	12	1,126	1.06	14	674	2.07	1.87 (0.86–4.06)	1.43 (0.53–2.93)
LAMAs								
Non use	337	10,217	3.29	437	7,616	5.73	1.67 (1.45–1.93)***	1.66 (1.44–1.93)***
Current use (≤30 d)	2	35	5.71	0	20	0	–	–
Recent use (30–90 d)	1	21	4.76	0	22	0	–	–
Past use (>90 d)	0	129	0	3	116	2.58	–	–
SABAs								
Non use	286	6,437	4.44	404	5.869	6.88	1.49 (1.28–1.73)***	1.61 (1.37–1.88)***
Current use (≤30 d)	6	345	1.73	6	212	2.83	1.61 (0.52–5.02)	1.98 (0.39–10.0)
Recent use (30–90 d)	3	211	1.42	4	105	3.80	2.61 (0.58–11.7)	32.2 (1.79–773.0)*
Past use (>90 d)	45	3,409	1.32	26	1,588	1.63	1.26 (0.78–2.05)	0.99 (0.58–1.67)
SAMAs								
Non use	312	8,187	3.81	410	6,457	6.34	1.60 (1.38–1.85)***	1.62 (1.39-1.89)***
Current use (≤30 d)	6	272	2.20	5	110	4.54	1.96 (0.59-6.49)	1.75 (0.27-11.0)
Recent use (30-90 d)	3	148	2.02	4	82	4.87	2.92 (0.64–13.2)	–
Past use (>90 d)	19	1,795	1.05	21	1,125	1.86	1.79 (0.96–3.33)	1.26 (0.62–2.52)
ICSs								
Non use	313	7,777	4.02	409	6,461	6.33	1.52 (1.31–1.76)***	1.52 (1.31–1.78)***
Current use (≤30 d)	0	210	0	3	139	2.15	–	–
Recent use (30–90 d)	1	100	1	1	60	1.66	2.55 (0.11–58.5)	–
Past use (>90 d)	26	2,315	1.12	27	1,114	2.42	2.13 (1.24–3.65)**	1.85 (1.01–3.39)*
OSs								
Non use	300	5,011	5.98	410	4,360	9.40	1.52 (1.31–1.76)***	1.52 (1.30–1.77)***
Current use (≤30 d)	6	727	0.82	8	628	1.27	1.74 (0.57–5.34)	2.12 (0.51–8.72)
Recent use (30–90 d)	3	512	0.58	6	439	1.36	2.53 (0.62–10.3)	2.13 (0.30–15.0)
Past use (>90 d)	31	4,152	0.74	16	2,347	0.68	0.91 (0.50–1.67)	0.88 (0.46–1.66)
Anti-arrhythmia								
Non use	330	9,998	3.30	433	7,618	5.68	1.66 (1.43–1.91)***	1.68 (1.45–1.94)***
Current use (≤30 d)	1	34	2.94	1	36	2.77	0.76 (0.04–12.9)	–
Recent use (30–90 d)	0	19	0	0	0		–	–
Past use (>90 d)	9	351	2.56	6	120	5	2.10 (0.74–5.94)	31.1 (3.25–297.8)**

*p < 0.05, **p < 0.01, ***p < 0.001.

HR is adjusted for sex, age, sleep disturbance, pulmonary tuberculosis, pneumonia, asthma, diabetes mellitus, hypertension, hyperlipidemia, pulmonary embolism, LABAs-use, LAMAs-use, SABAs-use, SAMAs-use, ICSs-use, OSs-use, and anti-arrhythmia drug use.

–– Unable to calculate because of few or no events.

As indicated in **Table 4**, a low dose of SABAs (< 1 DDD), medium dose of SABAs (1–20 DDD), low dose of SAMAs (< 2 DDD), low dose of ICSs (< 250 DDD), medium dose of ICSs (250–420 DDD), low dose of OSs (< 4 DDD), and medium dose of OSs (4–14 DDD) were significantly associated with a 2.12-fold (95% CI = 1.46–3.10), 3.48-fold (95% CI = 1.13–10.6), 3.19-fold (95% CI = 2.04–4.99), 28.1-fold (95% CI = 14.2–555.7), 2.09-fold (95% CI = 1.32–3.29), 2.28-fold (95% CI = 1.53–3.40), and 1.93-fold (95% CI = 1.36–2.74) higher risk of HDS in the BCOS cohort, respectively, relative to the non-BCOS cohort.

**Table 4:** Incidence rate and hazard ratio of stroke or heart-disease between the two groups stratified by cumulative drug dosage after propensity matching.

	Bronchiectasis - COPD						Crude HR (95% CI)	Adjusted HR (95% CI)
	No			Yes				
	Event	PY	IR	Event	PY	IR		
**Cumulative dose of drug**								
LABAs (DDD) (µg/d)								
Non-use	323	8,993	3.59	404	6,855	5.89	1.58 (1.36–1.83)***	1.62 (1.39–1.88)***
<210	3	595	0.50	11	227	4.84	9.78 (2.69–35.5)***	–
210–420	14	814	1.71	22	677	3.24	1.83 (0.93–3.60)	1.11 (0.50–2.44)
>420	0	0		3	15	20	–	–
LAMAs (mg/d)								
Non-use	335	10,201	3.28	432	7,587	5.69	1.67 (1.44–1.93)***	1.68 (1.45–1.94)***
<1	2	82	2.43	0	32	0	–	–
1–30	0	0		0	0		–	–
>30	3	119	2.52	8	155	5.16	2.26 (0.59–8.64)	2.01 (0.42–9.54)
SABAs (mg/d)								
Non-use	263	6,329	4.15	334	5,630	5.93	1.37 (1.16–1.61)***	1.56 (1.31–1.84)***
<1	54	2,538	2.12	70	1,243	5.63	2.62 (1.83–3.74)***	2.12 (1.46–3.10)***
1–20	8	498	1.60	14	220	6.36	4.04 (1.68–9.74)**	3.48 (1.13–10.6)*
>20	15	1,037	1.44	22	681	3.23	2.20 (1.14–4.25)*	1.73 (0.78–3.85)
SAMAs (mg/d)								
Non-use	296	8,097	3.65	353	6,281	5.62	1.48 (1.26–1.72)***	1.53 (1.31–1.80)***
<2	38	1,883	2.01	51	812	6.28	3.08 (2.02–4.69)***	3.19 (2.04–4.99)***
2–20	0	37	0	2	18	11.1	–	–
>20	6	385	1.55	34	663	5.12	3.15 (1.32–7.52)**	2.31 (0.75–7.15)
ICSs (µg/d)								
Non-use	302	7,714	3.91	372	6,340	5.86	1.44 (1.24–1.68)***	1.53 (1.31–1.79)***
<250	2	508	0.39	7	184	3.80	9.13 (1.88–44.1)**	28.1 (1.42–555.7)*
250–420	36	2,120	1.69	59	1,239	4.76	2.73 (1.80–4.15)***	2.09 (1.32–3.29)**
>420	0	60	0	2	11	18.1	–	–
OSs (mg/d)								
Non-use	184	4,344	4.23	241	3,716	6.48	1.44 (1.19–1.74)***	1.57 (1.29–1.92)***
<4	54	2,385	2.26	60	1,038	5.78	2.53 (1.75–3.67)***	2.28 (1.53–3.40)***
4–14	57	2,345	2.43	85	1,897	4.48	1.81 (1.30–2.54)***	1.93 (1.36–2.74)***
>14	45	1,328	3.38	54	1,123	4.80	1.39 (0.93–2.07)	1.34 (0.84–2.14)
Anti-arrhythmia (mg/d)								
Non-use	325	9,980	3.25	431	7,612	5.66	1.67 (1.45–1.93)***	1.69 (1.45–1.96)***
<30	0	0		0	0		–	–
30–35	11	366	3.00	7	114	6.14	2.12 (0.80–5.62)	3.95 (0.83–18.6)
>35	4	57	7.01	2	48	4.16	0.59 (0.10–3.50)	–

COPD, chronic obstructive pulmonary disease; DM, diabetes mellitus; PY, person-years; IR, incidence rate, per 1,000 person-years; HR, hazard ratio; CI, confidence interval; *p < 0.05, **p < 0.01, ***p < 0.001.

HR is adjusted for gender, age, sleep disturbance, pulmonary tuberculosis, pneumonia, asthma, diabetes mellitus, hypertension, hyperlipidemia, pulmonary embolism, LABAs-use, LAMAs-use, SABAs-use, SAMAs-use, ICSs-use, OSs-use, and anti-arrhythmia drugs use.

– Unable to calculate because of few or no events.

**Figure 1:**
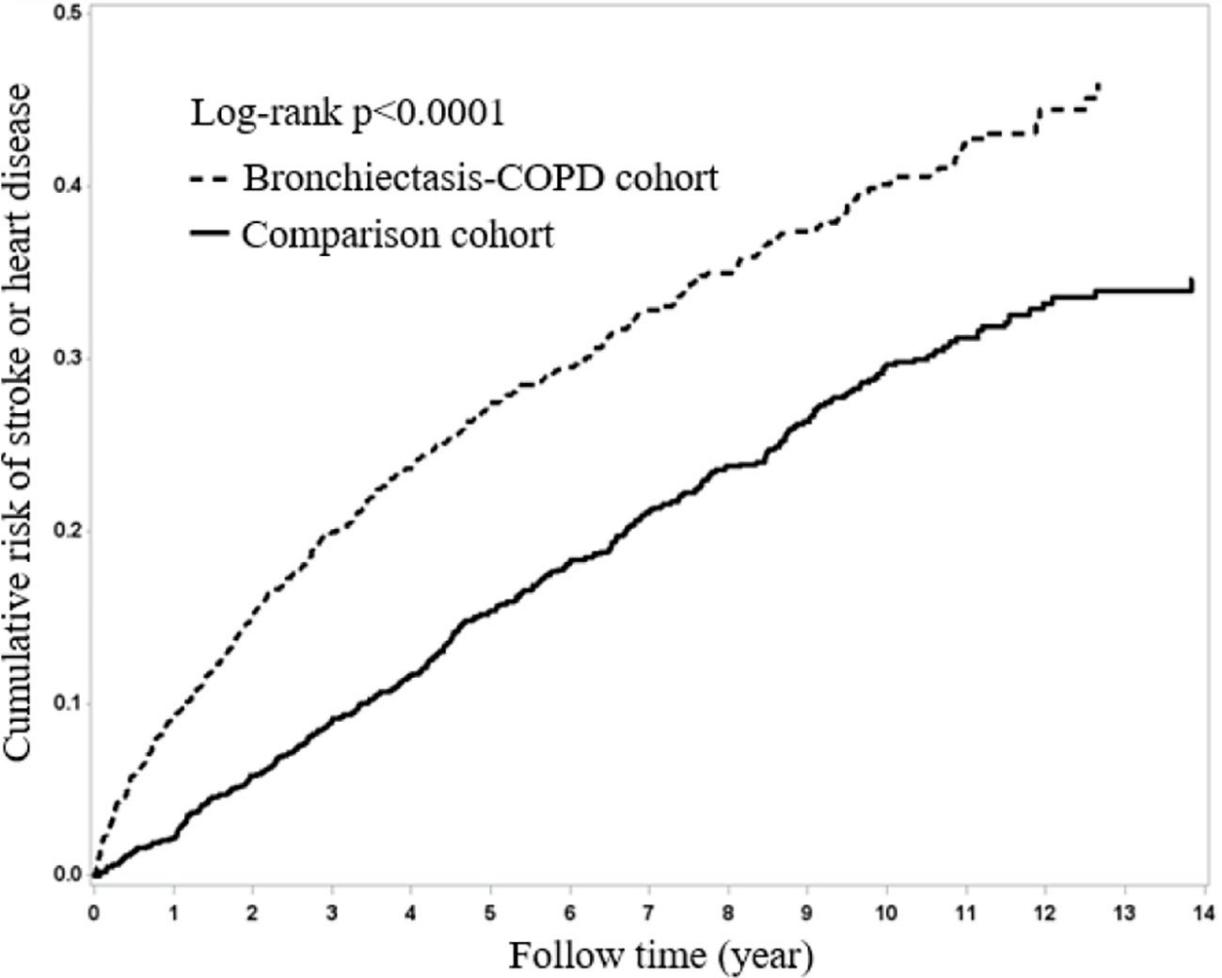
Kaplan–Meier curves of overall survival for the patients both with and without bronchiectasis-COPD (log-rank p < 0.0001).

**Figure 1** presents Kaplan–Meier curves for HDS risk stratified by the two cohorts and compared using a log-rank test. BCOS was associated with a significantly increased risk of HDS events (log-rank P < 0.001). From the first year of follow-up to the end of the study period, the incidence of HDS events in the BCOS cohort was higher than that in the non-BCOS cohort.

### Indication and Healthy User Bias

In this study, we analyzed LABAs/LAMAs, SABAs/SAMAs, ICSs/OSs and anti-arrhythmia drugs use in the BCOS cohort to determine the effect of these drugs on the risk of HDS in this population. We excluded all patients with a history of bronchiectasis and COPD diagnosis and drug use before the diagnosis of BCOS. Accordingly, all patients included in the BCOS cohort were new users of the mentioned drugs. We classified these patients according to drug use days into the following subgroups before HDS incidence: current, recent, and past. Thus, we could analyze the effects of the drugs on the current, recent, and past users in the BCOS cohort. Furthermore, BCOS severity may be a confounding factor of drug use, with patients with more severe BCOS receiving higher drug doses. To resolve this problem, we analyzed the DDD to determine how different doses of a drug may affect the risk of HDS (Yeh et al., 2018b).

## Discussion

This general population study resulted in four major findings. First, patients with BCOS, even those without comorbidities, had a higher risk of HDS. Martinez-Garcia et al., 2018 Second, recent SABAs use, past ICSs use, and past anti-arrhythmia drugs use were associated with a relatively high risk of HDS. Third, a low to medium dose of SABAs, ICSs, or OSs and a low dose of SAMAs were associated with a relatively high risk of HDS. Fourth, LAMAs were not significantly associated with HDS risk.

Wang et al. (2018) reported that the initiation (< 30 days) of LABAs or LAMAs in patients with COPD was associated with an approximately 1.5-fold increase in severe cardiovascular risk, irrespective of prior HDS status or history of exacerbation. In the present study, we found that LAMAs were not associated with HDS. Evidence from observational studies supports the possible link between the use of LABAs/LAMAs for COPD management and HDS, although confounding and bias cannot be entirely ruled out for the positive findings. Moreover, although randomized controlled trials (RCTs) have not documented that LABAs/LAMAs therapy in patients with COPD is associated with an elevated risk of HDS, null findings may result from insufficient statistical power, the exclusion of patients at a high risk of HDS, and failure to account for the use of other respiratory medications during follow-up. The role of LABAs/LAMAs in the risk of HDS among patients with BCOS remains controversial (Wang et al., 2019). Future RCTs and observational studies are urgently required to address the noted limitations and clarify how these drugs affect HDS risk.

Recent use and low or medium doses of SABAs are associated with a higher risk of HDS. According to one previous study (Macie et al., 2008), SABAs administration resulted in hypoxemia, a paradoxical reaction (Taylor and Hancox, 2000); this paradoxical reaction may explain the higher risk of HDS associated with SABAs use (Fan et al., 2016). Hypoxemia was also associated with an increased wall-area percentage of segmental airways (odds ratio [OR] = 1.04, 95% CI = 1.01–1.08), in addition to being independently associated with aggravated dyspnea, a shorter 6-min walking distance, a higher BODE (Body mass index, airflow Obstruction, Dyspnea, and Exercise) index, and a greater frequency of exacerbation (Bhatt et al., 2014). BCOS is a disorder combined with these poor indices. Consequently, the inappropriate use of SABAs may aggravate hypoxemia and promote HDS development (Fan et al., 2016).

Bronchiectasis and COPD are airway and system inflammation diseases. BCOS is a disorder that combines both of these diseases. Thus, patients with BCOS have a higher level of system inflammation (Coban and Gungen, 2017), whic corresponds to poor lung function and quality score (Chen et al., 2015; Dos Santos et al., 2018). This high level of inflammation combined with hypoxemia contributes to the atherosclerosis of the arteries in patients with BCOS, meaning that atherosclerosis-related HDS incidents (Gao et al., 2018b) occur at a higher rate in patients diagnosed as having both bronchiectasis (Gao et al., 2018a) and COPD. The limited anti-inflammatory effects (Keränen et al., 2016; Koarai and Ichinose, 2018) of LABAs/LAMAs, SABAs/SAMAs, and ICSs/OSs means that these drugs may have difficulty ameliorating the progress of persistent arterial atherosclerosis. However, in this study, a high DDD of OSs was not associated with HDS risk. One possible explanation for this is that patients exhibiting the eosinophilic phenotype of BCOS may have favorable responses to these drugs, which may thus attenuate the risk of HDS (Everaerts et al., 2018).

Previous studies have found that use of OSs, whether current, recent, or past, did not coincide with a high risk of HDS. However, low to medium doses of OSs may be associated with a high risk of HDS. BCOS is a disorder with an immunocompromised component, which aggravates immunodeficiency. The system effect of OSs contributes to hyperglycemia, hypertension, and hyperlipidemia development, which are predisposing factors for HDS. Thus, OSs use is associated with HDS development, as discovered in the present study. Accordingly, steroids should not be routinely used in BCOS treatment (Flume et al., 2018).

We also found that although anti-arrhythmia drugs use were generally associated with a higher risk of HDS, a medium DDD of 30–36 did not increase the risk of HDS. This is consistent with the finding of Chen et al. (2017a; 2017b) that anti-arrhythmia drugs used as rhythm or rate control agents in arrhythmia treatment were associated with a reduction in cardiovascular risk. These findings imply that past or optimal doses of anti-arrhythmia drugs may attenuate the risk of incident HDS.

Finally, patients with BCOS have a poor prognosis (De Soyza et al., 2017); hence, such patients, particularly those who frequently use SABAs, SAMAs, or OSs in the later course of BCOS treatment, have higher mortality rates compared with other patients (Fan et al., 2016). However, because these patients have a shorter life-spans, they are also at a lower risk of HDS. Therefore, past use or high doses of SABAs (aHR, 1.73; OR, 0.78–3.85), SAMAs (aHR, 2.31; OR, 0.75–7.15), or OSs (aHR, 1.34; OR, 0.84–2.14) were not associated with HDS. This finding may represent an underestimation of the risk of HDS in these groups. Moreover, patients who frequently use these drugs are more likely to strictly adhere to the medical directions provided for them, which may also serve to limit the incidence of HDS (Yeh et al., 2019b). This implies that LAMAs; optimal time; and the dose of SABAs, SAMAs, or OSs in patients with mild to moderate BCOS may provide protective effects against acute incident HDS. However, this speculation warrants further research.

Although RCTs can produce valuable information, they are associated with several drawbacks: they require a substantial amount of money, results obtained from animal experiments are not completely applicable to humans, and human trials can have considerable ethical complications in the real world. For example, we cannot routinely prescribe the long-term use of anti-arrhythmia drugs to patients with BCOS without consulting an expert (e.g., a cardiologist). Currently, the long-term prescription of ICSs and LABAs is strictly controlled by cardiologists in Taiwan.

Furthermore, the new LAMAs added on the ICSs/LABAs associated with the increased risk of HDS among COPD cohort in the finding of the Liou et al. (2018). Our study revealed that recent use of SABAs was associated with the higher risk of HDS and LAMAs were not associated with the HDS. These findings alert the physician to detect the risk of the HDS in the recent use of SABAs and encourage the physician to early use of the LAMAs among the BCOS cohort. Meanwhile, the frequency of the BCOS cohort receiving the LAMAs was higher than the non-BCOS cohort in this study.

The comparision cohort (non-BCOS) including the ACO. Therefore, the non-BCOS cohort having the higher frequency of receiving the medicine such as LABAs, SABAs, SAMAs, and ICSs/OSs than the BCOS cohort (Yeh et al., 2018a; Yeh et al., 2018b; Yeh et al., 2019b).

This large-scale study explored the effect of bronchodilator, ICSs/OSs, and anti-arrhythmia drugs use on the risk of HDS in the BCOS cohort based on the large scale. Our results may serve as a reference for the future research (e.g., RCTs) of the BCOS. Thus, if the BCOS is considered as a different separate entity disease; our study may offer useful information for providing the appropriate and personalized medicine in patients with BCOS. (**Appendix Figure 1**)

### Strengths

The accuracy of the medical records in the NHIRD is high; therefore, this database is a valuable resource for population research on cardiovascular disease and stroke (Cheng et al., 2014). Ho et al. (2018) validated the COPD cohort from the NHIRD. Thus, the BCOS cohort extracted from the NHIRD is reasonable. Moreover, the NHIRD-based identification of COPD (Ho et al., 2018) and bronchiectasis-related diseases, such as pulmonary tuberculosis and pneumonia, has been validated in several recent reports (Ho et al., 2018; Yeh, 2018; Yeh, 2018b). Therefore, the use of this well-established database limits the potential bias in this study. We also carefully followed up each of the patients studied (for 5.51 ± 4.02 years for the patients in the BCOS cohort and for 7.79 ± 4.2 years for the patients in the non-BCOS cohort). Our long-term follow-up process may be more beneficial than that performed in RCTs, particularly when studying older patients or patients who have multiple comorbidities (Booth and Tannock, 2014). As mentioned, we analyzed the effects of treatment duration and dose on the risk of HDS. Our methods may help avoid the confounding factors typically found in cohort studies.

### Limitations

Possible limitations of this study are biases and confounding variables, such as choice of exposure risk window or confounding by indication. Risk windows can be validated or a sensitivity analysis may be conducted based on varying lengths of exposure risk windows—here, we analyzed the DDD of the drugs examined. For confounding by indication, randomization helps to prevent selection bias by a clinician, and we included the frequency of outpatient or inpatients visits. Another potential limitation is protopathic bias, which can be controlled by adding a lag time to the exposure (e.g., excluding exposure that occurred in a period before the outcome within HDS). We analyzed the risk of HDS in patients within 30 to 90 days and >90 days of drug use. Finally, surveillance bias is also a concern. Evidence-based clinical guidelines should specify which at-risk patients must be studied and should clearly convey exact testing modalities and frequencies. In our study, we analyzed patients with and without comorbidities (Levesque et al., 2010; Hajian Tilaki, 2012).

We used several designs and factors to control for bias and confounding variables: new user design (entry after cohort), case-only design (past, recent, current), and disease risk score (frequency of outpatient and inpatient visits). Within this framework, these methods are similar to those utilized in RCTs. However, our results are not as accurate as those that would have been obtained from an RCT (Hajian Tilaki, 2012). In addition, cytokine data are unavailable in the NHIRD. We used a new user approach to evaluate healthy user bias. However, data on lifestyle changes (e.g., exercise and diet) in low- or high-adherence patients are unavailable in the NHIRD; these confounding factors may have led to some biases in the present study.

Our asthma–COPD–bronchiectasis subcohort was derived from the COPD cohort of the NHIRD. Because the treatment principle of this subcohort was observed to be similar to that of the bronchiectasis–COPD subcohort (Bell et al., 2018), we enrolled the bronchiectasis–COPD subcohort as the BCOS cohort and considered the asthma–COPD–bronchiectasis cohort as a distinct entity within the BCOS cohort. Thus, we may list this subcohort as another confounding factor of our study.

## Conclusion

Compared with patients without bronchiectasis–COPD, patients with BCOS with recent SABAs use, past ICSs use, past anti-arrhythmia drugs use, a low or medium SABAs dose, a low or medium ICSs dose, a low or medium OSs dose, and a low SAMAs dose had a higher risk of HDS. LAMAs were not associated with HDS risk.

## Data Availability Statement

The dataset used in this study is held by the Taiwan Ministry of Health and Welfare (MOHW). The Ministry of Health and Welfare must approve our application to access this data. Any researcher interested in accessing this dataset can submit an application form to the Ministry of Health and Welfare requesting access. Please contact the staff of MOHW (Email: stcarolwu@mohw.gov.tw) for further assistance. Taiwan Ministry of Health and Welfare Address: No.488, Sec. 6, Zhongxiao E. Rd., Nangang Dist., Taipei City 115, Taiwan (R.O.C.). Phone: +886-2-8590-6848. All relevant data are within the paper.

## Ethics Statement

The NHIRD encrypts patient personal information to protect privacy and provides researchers with anonymous identification numbers associated with relevant claims information, including sex, date of birth, medical services received, and prescriptions. Therefore, patient consent is not required to access the NHIRD. This study was approved to fulfill the condition for exemption by the Institutional Review Board (IRB) of China Medical University (CMUH-104-REC2-115-CR3). The IRB also specifically waived the consent requirement.

## Author Contributions

All authors contributed significantly, and all authors agree with the manuscript content. Conception/Design: J-JY, C-HK. Provision of study materials: C-HK. Collection and/or assembly of data: All authors. Data analysis and interpretation: All authors. Manuscript writing: All authors. Final approval of manuscript: All authors.

## Conflict of Interest

The authors declare that the research was conducted in the absence of any commercial or financial relationships that could be construed as a potential conflict of interest.

## Abbreviations

HDS, heart disease/stroke; BCOS, bronchiectasis–chronic obstructive pulmonary disease overlap syndrome; COPD, chronic obstructive pulmonary disease; aHR, adjusted hazard ratio; CI, confidence interval; LABA, long-acting β2-agonist; LAMA, long-acting muscarinic antagonist; SABA, short-acting β2-agonist; SAMA, short-acting muscarinic antagonist; OS, oral steroid; ICS, inhaled corticosteroid steroid; HRCT, high-resolution computed tomography; NHIRD, National Health Insurance Registration Database; ICD-9-CM, International Classification of Diseases, Ninth Revision, Clinical Modification; Cumulative defined daily dose, DDD.
