# Supplementary figures and images for: Effect of Bronchodilator and Steroid Use on Heart Disease and Stroke Risks in a Bronchiectasis–Chronic Obstructive Pulmonary Disease Overlap Cohort: A Propensity Score Matching Study
Source: Front Pharmacol. 2019 Nov 27;10:1409. doi: 10.3389/fphar.2019.01409 (PMC6895570; doi:10.3389/fphar.2019.01409)

Appendix figure 1. BCOS as a different separate entity disease.

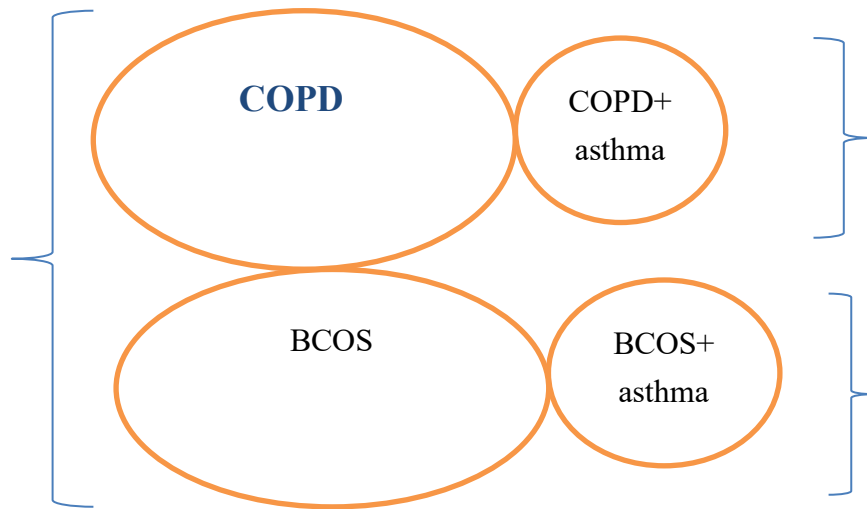

Supplement: Supplementary file 1 [file Image_1.pdf]
